# Development of a Vitrification Preservation Process for Bioengineered Epithelial Constructs

**DOI:** 10.3390/cells11071115

**Published:** 2022-03-25

**Authors:** Lia H. Campbell, Kelvin G. M. Brockbank

**Affiliations:** Tissue Testing Technologies LLC, 2231 Technical Parkway, Suite A, North Charleston, SC 29406, USA; kgbrockbankassoc@aol.com

**Keywords:** vitrification, bioengineered tissue constructs, cryopreservation

## Abstract

The demand for human bioengineered tissue constructs is growing in response to the worldwide movement away from the use of animals for testing of new chemicals, drug screening and household products. Presently, constructs are manufactured and delivered just in time, resulting in delays and high costs of manufacturing. Cryopreservation and banking would speed up delivery times and permit cost reduction due to larger scale manufacturing. Our objective in these studies was development of ice-free vitrification formulations and protocols using human bioengineered epithelial constructs that could be scaled up from individual constructs to 24-well plates. Initial experiments using single EpiDerm constructs in vials demonstrated viability >80% of untreated control, significantly higher than our best freezing strategy. Further studies focused on optimization and evaluation of ice-free vitrification strategies. Vitrification experiments with 55% (VS55) and 70% (VS70) cryoprotectant (CPA) formulations produced constructs with good viability shortly after rewarming, but viability decreased in the next days, post-rewarming in vitro. Protocol changes contributed to improved outcomes over time in vitro. We then transitioned from using glass vials with 1 construct to deep-well plates holding up to 24 individual constructs. Construct viability was maintained at >80% post-warming viability and >70% viability on days 1–3 in vitro. Similar viability was demonstrated for other related tissue constructs. Furthermore, we demonstrated maintenance of viability after 2–7 months of storage below −135 °C.

## 1. Introduction

The ongoing demand to reduce the number of animals being used in research continues to drive the development of in vitro assays, both cell- and tissue-based assays, that provide accurate toxicity data about various chemicals and compounds. In 2009, the European Union banned the use of animals for testing of cosmetic ingredients. This ban has increased the demand for in vitro predictive assays and it is anticipated that the rest of the world will follow, particularly if they wish to market cosmetic products in Europe. The US, as a member of the Organization for Economic Cooperation and Development (OECD), has accepted EpiDerm and several other tissue models for skin corrosion and skin irritation testing (OECD TG 431 and 439, respectively). These tests are being used by many companies, even though it is not illegal to use animals in the US. It is anticipated that a ban of the use of animals for toxicity testing of other types of compounds, including pharmaceuticals and household chemicals, will soon follow, and there is an increasing amount of research that supports the use of tissue-engineered constructs from a variety of tissues, not just skin, for toxicology testing. Companies involved in the production of cosmetics, chemicals, household products and pharmaceuticals have started using tissue equivalent constructs [1,2,3,4,5,6,7,8] and it is anticipated that engineered tissue models will eventually replace many in vivo preclinical animal tests [9].

Currently, tissue constructs such as EpiDerm from MatTek are made to order, so a lead time of several weeks is required to manufacture them prior to being shipped. Shipments are sent overnight at 4°C and the constructs must be used within a short time period (1–2 weeks) for the best results. Furthermore, quality-control testing cannot be completed prior to shipment, due to the short time that these skin equivalents can be used, but must be carried out after receipt by the customer. This may result in the rejection of data from these batches when the customer has already expended extensive resources, time and effort using these constructs. Availability can also be an issue if a validated construct becomes unavailable for various reasons, such as weather or production problems. Then, development of drugs and other compounds are delayed, while time and money are wasted due to inactivity. A method to preserve these constructs would eliminate the lead time required to make the constructs in response to orders, allow quality control checks for stock prior to shipping and reduce costs due to economies of scale. The end user would have greater flexibility for experiment scheduling without concerns about construct availability or quality. They can also order large quantities of a specific product batch in order to better control variability due to the test constructs within studies.

Cryopreservation of most cells in suspension is quite routine [10]. However, the cryopreservation of more complex tissues and organs is neither routine nor very well developed. It is not necessarily the ability to cryopreserve the cells that is the more difficult task, rather it is successfully cryopreserving the cells within the culture system required for the tissue constructs; in this case, a multi-well plate plus well insert. Then, there is the additional challenge of successfully cryopreserving a multi-cell layer construct within this complex culturing system without disruption of the construct. To date, there has been no successful protocol for cryopreserving whole organs, short-term storage at 4–6 °C for hours or under normothermic physiological conditions is all that has been achieved for human organ transplantation. Some tissues, such as donor-derived skin grafts, are routinely cryopreserved. However, the transplanted keratinocytes or sheets are not expected to survive, because of the allogeneic source of the cells, but they are clinically effective (i.e., ApliGraft manufactured by Organogenesis, Inc., Canton, MA, USA) and they promote healing by releasing cytokines that trigger the migration and proliferation of the native graft recipient keratinocytes [11,12]. Long-term survival of the cells is not expected or required. In order for cryopreserved epidermal models and other tissue constructs to have true value, they must be successfully cryopreserved and thawed demonstrating good viability and function for at least several days post-thaw. Additionally, the ability of the cells or monolayers to remain intact and attached to their substrate is absolutely required to maintain the structural integrity and function required of tissue models.

While cryopreservation has been used for many years, development of processes that can preserve complex tissues and organs has remained elusive, but vitrification could provide a suitable alternative. Vitrification is the solidification of a liquid without crystallization and can be achieved by adjusting the solute composition and cooling rate [13,14,15]. Vitrification has been shown to provide effective preservation for some cells, specifically cells for reproduction, such as sperm, oocytes and embryos [16,17]. More recently, we have developed methods for vitrification of more complex tissues in which freezing methods have not proven to be very effective, such as blood vessels, articular cartilage and heart valves [18,19,20,21,22,23,24,25,26,27,28,29,30,31,32,33]. These relatively thin complex tissues [22,23,26,27,29,30,31,32] have been successfully vitrified with excellent retention of cell viability and in vivo function [19,20,24,29,33] without disrupting their overall architecture or extracellular matrix [18,21,24,29].

In this study, a vitrification protocol was developed to preserve human bioengineered skin-equivalent constructs, EpiDerm. Successful preservation of the proposed constructs was carried out by combining a two-step warming strategy with cryopreservation by ice-free vitrification. Optimization of cryoprotectant loading with minimization of cytotoxicity was performed. Due to our past experience and development of the CryoPlate preservation strategy employing corneal endothelial cell monolayers, and vitrification of tissues [34,35,36,37,38], we were in a unique position to develop strategies for more complex engineered products, particularly in multi-well plates, because we had already begun to address the questions of maintenance of cell viability and attachment to substrates [34,35,39,40,41,42].

## 2. Materials and Methods

### 2.1. Construct Maintenance

Constructs were obtained from MatTek (www.mattek.com, accessed on 22 March 2022) and maintained according to the manufacturer’s specifications. All constructs were incubated in appropriate media for 24 h under physiological tissue culture conditions before initiating experiments. Fresh controls were used in each experiment and both fresh and experimental treatment groups were assessed up to 4 days in vitro post-rewarming.

### 2.2. Vitrification Methods

The basic protocol for vitrifying constructs is described. Modifications were made based on what type of tissue was being vitrified, the volume being used, and the vessel or container used for vitrification. The construct was gradually infiltrated with precooled vitrification formulations at 4 °C in 5 min incubation increments consisting of 0%, 12.5%, 25%, 50%, 75% and 100% of each formulation to achieve a final cryoprotectant concentration. The constructs were placed into either glass scintillation vials (Diam. *x* H, 25 mm × 60 mm) containing 1.5 mL of pre-cooled vitrification solution with 0.3 mL solution inside the well insert or 24-well deep-well plates (Height 47 mm, 125 mm, 84 mm) (Thomson Instrument Co., Oceanside, CA, USA, #931568), and 0.6 mL of pre-cooled vitrification solution was added to the well with 0.2 mL solution inside the well insert. Samples were cooled rapidly (~45 °C /min) to −100 °C by placing the samples in a pre-cooled bath containing isopentane in a −135 °C mechanical storage freezer (Revco, Thermo Fisher, Waltham, MA, USA). Upon achieving −100 °C, the specimens were removed from the bath and stored at −135 °C in the bottom of the mechanical storage freezer, which results in slow cooling (3 °C /min) to −135 °C. The samples were held at −135 °C for a minimum of 24 h. The constructs were rewarmed in two stages, first, slow warming to −100°C (~30 °C /min) at the top of the mechanical storage freezer and then rapidly warmed to either 0 °C or ±−10 °C (~225 °C /min) in a 30% ME_2_SO bath at room temperature. After rewarming, the vitrification solution was removed in 7 sequential 5 min steps at 4 °C into culture medium, as previously described [20,22,26,29,31,32,43].

### 2.3. Cryopreservation by Freezing Methods

Constructs were incubated on ice with 15% Glycerol-10%HES (200 µL in insert + 500 µL in the well) in a 24-well culture plate for 20 min; then, all constructs were transferred to a new 24-well plate with fresh 15% Glycerol-10%HES (200 µL in insert + 500 µL in the well); then cooled to −80 °C using a controlled rate freezer using a modified cooling rate of −1 °C /min with a nucleation step included at ~−6 °C. The plate was then stored in vapor-phase nitrogen until thawing. The thawing process involved transferring the plate to −20 °C for 30 min and then rewarming rapidly using a 37 °C water bath. This included addition of warmed (37 °C) 0.5 M mannitol to each well before placing the plate on ice. Constructs were transferred to a 12 well plate and 4 mL 0.5 M mannitol was used to wash the constructs and remove the cryoprotectant. Constructs were then moved to a 24-well plate in culture media and were allowed to recover for 60 min at 37 °C before metabolic activity was measured [34,35,39,40,41].

### 2.4. MTT Assay

This assay measures metabolic activity and is included because it is the most common assay used for assessment of skin-equivalent viability. The MTT [3-(4,5-dimethylthiazol-2-yl)-2,5-diphenyltetrazolium bromide] assay is based on the ability of a mitochondrial dehydrogenase enzyme from viable cells to cleave the tetrazolium rings of the pale-yellow MTT to form dark-blue formazan crystals that are largely impermeable to cell membranes, thus resulting in its accumulation within healthy cells. Solubilization of the cells by the addition of a detergent results in the liberation of the crystals and cell viability is directly proportional to the level of the formazan product created. The color can then be quantified using a simple colorimetric assay on an absorbance reader at 570 nm (Molecular Devices, San Jose, CA, USA).

### 2.5. Resazurin Assay

The resazurin assay assesses viability of the constructs by measuring the oxidation/reduction reactions that take place within cells in the construct. Resazurin dye was added directly to the culture wells at 10% of the assay volume (i.e., 30 µL resazurin in 300 µL culture medium) and constructs were incubated for 3 h at 37 °C. Upon reduction, the dye changes color and this color change will be measured and quantified. Plates were read using the Fmax fluorescent microplate reader (Molecular Dynamics, Sunnyvale, CA, USA) at an excitation wavelength of 544 nm and an emission wavelength of 590 nm. This assay is non-toxic, so constructs can be assessed before and several times after treatment, each construct can be its own control and allowed for detection of cell proliferation and delayed cell death in the constructs. Measurement of viability using the resazurin assay correlates well with the MTT assay, which is the assay of choice for measurement of viability in toxicity testing (correlation coefficient R^2^ = 0.99).

### 2.6. IL-1α Release

IL-1α is an important regulator of immune and inflammatory responses. It is used in addition to the MTT assay to measure and predict the irritancy of substances being tested. IL-1α is released into the supernatant, and so the conditioned media samples from fresh and cryopreserved tissue constructs were saved and the IL-1α concentration measured using an EIA assay (R&D Systems, Minneapolis, MN, USA).

### 2.7. Dose Response Assay

(Functional End-Point Assay employed by MatTek.) The dose response assay was performed according to the manufacturer’s instructions. Fresh tissues were used the following day, and treated groups were vitrified. The test begins with application of 100 μL of 1% TritonX-100 at time intervals of 4, 6, 8 and 12.5 h. After Triton exposure, tissue constructs were rinsed with sterile PBS followed by immediate transfer to an MTT assay for assessment of construct viability [44].

### 2.8. Histological Assessment

Fresh construct samples and select samples that were vitrified were fixed in 10% formalin and sent to AML Labs (Saint Augustine, FL, USA) for further processing and analysis. Samples were paraffin embedded, 5 µm sections were taken and stained with hematoxylin and eosin before being mounted and photographed.

### 2.9. Statistical Methods

All experiments were performed several times. The statistical analysis used was appropriate to the type of variable and the goal of the experiment. For measurable variables, *t*-test, analyses of variance (ANOVA, with multiple comparisons using Tukey’s, Bonferroni or Sheffe adjustment of a-error) and logistic regression methods was used.

## 3. Results

**Figure 1:**
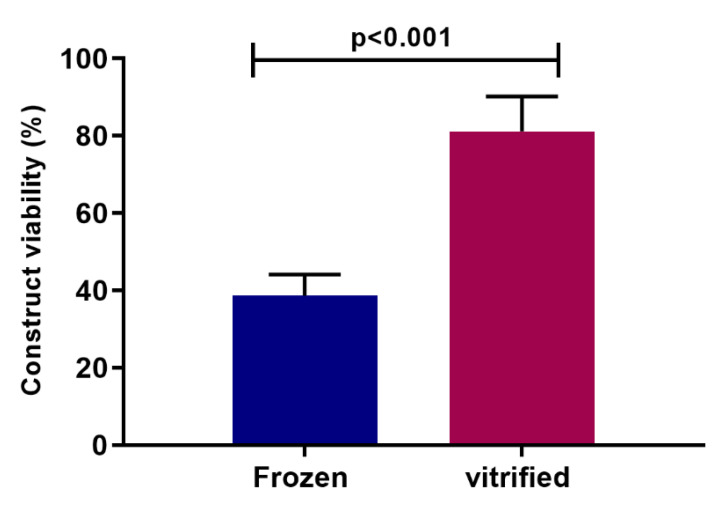
Viability of EpiDerm constructs after freezing and vitrification. Constructs were either cryopreserved by freezing in 15% glycerol/10% hydroxyethyl starch (HES) or vitrified in VS55. Viability was calculated as the mean (±SEM) of 9–12 replicates. Statistical comparison performed by T test.

The objective of this study was to develop and optimize a preservation strategy for tissue equivalents. Three-dimensional tissue constructs are becoming the alternative strategy for testing new products, chemicals and therapeutics, as they are more indicative of the in vivo environment, while reducing the number of animals used in research. Initial experiments were performed to develop a cryopreservation strategy by freezing for the tissue construct—EpiDerm from MatTek. A freezing protocol was used that we had developed for cryopreserving cell monolayers in multi-well plates [34,35,39,40,41]. Several different cryopreservation solutions were evaluated. Some were derived from the literature [45,46], such as 10% glycerol/20% fetal calf serum, and some were solutions we had used successfully with other cell types. These included 1 M DMSO in Unisol, 2 M propylene glycol in Euro-Collins and 150 mM trehalose in Hepes-buffered saline. The best viability, which was <50% of untreated controls, was obtained using a combination of 15% glycerol and 10% hydroxyethyl starch (Figure 1). However, investigation of an alternative strategy, vitrification, demonstrated improvement in viability.

The first vitrification experiments employed single EpiDerm inserts and asked the question of whether a tissue construct on an insert could be successfully vitrified. Tissues that had been successfully vitrified in previous studies [18,19,20,21,22,24,25,26,27,28,29,30,31,32,33] did not include a well insert in the vitrification solution changing the dynamics of the rapid cooling and warming process that are hallmarks of successful vitrification. Viability was measured at >80% (Figure 1), indicating that vitrification of a construct in a well insert was possible and significantly better than our best preservation protocol by freezing (*p* < 0.001).

**Figure 2:**
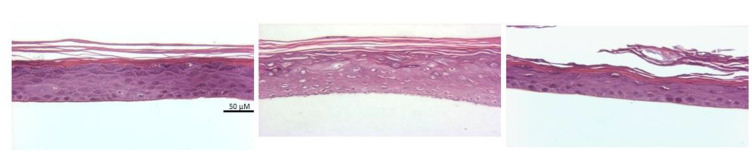
Histological evaluation of EpiDerm constructs. A fresh construct (**left**) was compared with a 15% glycerol–10% HES frozen construct (**middle**) and a VS55 vitrified construct (**right**). H&E-stained sections at 40×.

Histological analysis was carried out to evaluate morphological characteristics of constructs after preservation by freezing and vitrification (Figure 2). Constructs were fixed in 10% formalin and then stained with hematoxylin and eosin to evaluate their overall structure after cryopreservation. As seen in Figure 2, the representative fresh control has a compact cell layer and the stratum corneum is present and closely associated with the cells. In the frozen sample, the stratum corneum is not as tightly associated and there are many spaces within the cell layer, suggesting that ice was present during preservation. In contrast, vitrified sections showed little if any spaces within the cell layer and the strateum corneum is tightly associated with the cell layer, looking very similar to the fresh control. These results demonstrated that while providing better viability after preservation, vitrification also maintained structure and extracellular matrix composition of the EpiDerm constructs with little, if any, disruption to the tissue architecture as compared to the frozen construct. Based on the comparative metabolic and histology results (Figure 1 and Figure 2), vitrification provided better preservation for these bioengineered constructs than conventional cryopreservation by freezing. Further studies focused on optimization and evaluation of ice-free vitrification strategies.

**Figure 3:**
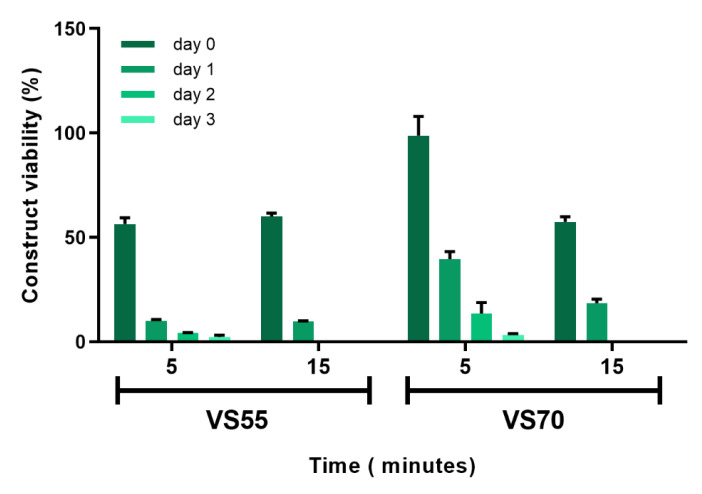
Viability of EpiDerm constructs after protocol modifications. Constructs were vitrified either using the original protocol for vein rings and segments that used 15 min load/unload steps with VS55 (8.4 M) [18,19,30,31,32,33] and another vitrification solution with a similar composition but higher final concentration, VS70 (10.7 M), or with shorter load/unload steps of 5 min. Viability was measured using the resazurin assay and calculated as the mean (±SEM) of 8 replicates. Statistical analysis performed by one-way ANOVA and Tukey’s multiple comparison test.

Repetitive vitrification experiments with VS55 and VS70 produced viable constructs with good viability shortly after rewarming, but viability decreased over days post-rewarming in vitro. In order for preserved constructs to have maximum value for testing applications, their ability to maintain viability for several days post-rewarming was considered important to allow more flexibility of use by the end user. Therefore, examination of the vitrification process, as well as evaluation of other vitrification solutions, was required to improve and sustain viability after rewarming. The initial vitrification experiments performed were carried out using a protocol developed for vein segments with the vitrification solution, VS55 containing 8.4 M CPAs [18,31,32,33,34]. This vitrification process used a 6-step (15 min incubation) load protocol to add CPA solution into the construct followed by vitrification in a glass vial in 1.5 mL vitrification solution with 0.3 mL solution inside the insert. The vials were cooled rapidly to −100 °C then slowly cooled to −135 °C where they were stored until rewarming (see methods section for details). Upon rewarming, the vitrification solution was removed using 7 sequential removal steps at 15 min each. Two adjustments were made that greatly improved viability. Incubation times during the load/unload steps were reduced to 5 min each and another vitrification solution was included, VS70, which produced better and more consistent viability then VS55. VS70 (10.7 M) contains the same components as VS55 (8.4 M), dimethyl sulfoxide (DMSO), propanediol (PD) and formamide (FD), but at different total concentrations. Logically, it would be assumed that VS55 should work better and cause less cytotoxicity because it has less overall CPAs as opposed to VS70; however, this was not what was observed. Both VS55 and VS70 produced similar viabilities using load/unload steps at 15 min (Figure 3). When the load/unload steps were reduced to 5 min, viability using VS55 did not significantly change from that observed using 15 min steps. However, constructs vitrified in VS70 using the shorter load/unload steps produced significantly better viability (*p* < 0.0001) (98%) than constructs vitrified with VS55 (39%). Additionally, while viability decreased over time in culture for both solutions using either load/unload time, viability was higher on consecutive days post-rewarming with VS70 then what was observed for VS55 (*p* < 0.0001, Figure 3).

One other small change to the protocol was made that improved consistency of the results, but did not improve viability for days post-rewarming. The first unload step was replaced with a simple dilution step that shortened the amount of time that the construct was exposed to the full-strength vitrification solution reducing potential cytotoxicity. While a significant improvement was not seen because of this protocol change, it was still included in the revised protocol because it did improve consistency across multiple samples.

**Figure 4:**
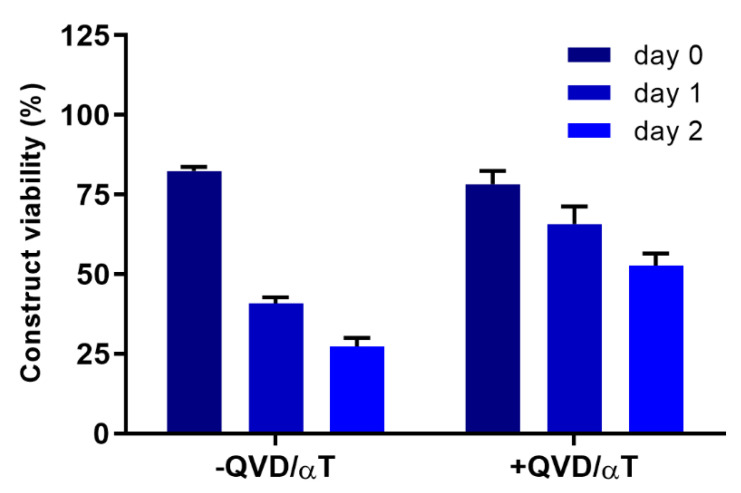
Construct viability after vitrification and recovery with and without additives. Constructs were vitrified in VS70 using our modified protocol. Constructs were incubated overnight in media with and without 100 µM α-tocopherol (αT) and 25 µM Q-VD-OPH (QVD), then vitrified in VS70. Upon rewarming, the constructs were incubated in media with or without QVD and αT for 2 days post-rewarming. Viability was measured using the resazurin assay and is the mean (±SEM) of >16 replicates. Statistical analysis performed by one-way ANOVA and Tukey’s multiple comparison test.

A viability of at least 60% is considered the minimum viability needed for an EpiDerm construct to be considered usable (personal communication, MatTek). Better viabilities are desirable and while these adjustments in the protocol improved viability immediately after rewarming with some improvement in viability for several days after rewarming, further changes were investigated to provide the high sustained viability for several days after rewarming that would be required for toxicity testing. The addition of the antioxidant α-tocopherol (αT) and the caspase inhibitor Q-VD-OPH (QVD) into the culture medium for the constructs before and after vitrification was one such adjustment (Figure 4). Constructs incubated overnight with QVD and αT prior to vitrification followed by inclusion of QVD and αT in the medium after rewarming were compared with constructs that were vitrified in VS70 without QVD and αT supplementation. The results demonstrated that viability for constructs supplemented with QVD and αT was similar to viability without supplementation immediately after rewarming. However, viability measured on consecutive days post-rewarming, days 1 and 2, demonstrated significant differences in viability between the two groups (*p* < 0.0001). The addition of QVD and αT maintained the viability of constructs for several days post-rewarming while the lack of supplementation produced the steady decrease in viability consistent with previous experiments. In addition to QVD and αT, other additives were evaluated for potential benefits to the vitrification process. These included ferulic acid, allene oxide synthase, curcumin, pluronic F68 and stromal-cell-derived factor 1 (SDF-1), but only QVD and αT demonstrated any significant improvement (data not shown).

**Figure 5:**
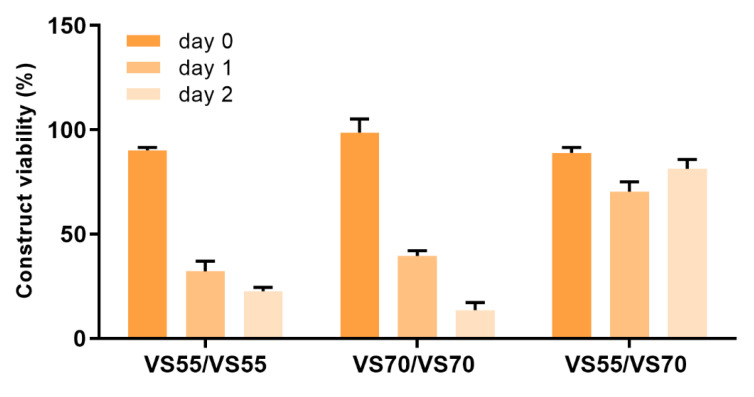
Construct viability using lower concentrations of load/unload solutions. Constructs were either loaded and unloaded with solutions based on the concentration of the vitrification solutions or the load/unload solutions used were at a lower concentration than the vitrification solutions. Viability was measured using the resazurin assay and is the mean (±SEM) of ≥8 replicates. Statistical analysis performed by one-way ANOVA and Tukey’s multiple comparison test.

The revised protocol was then employed for further exploration of the vitrification protocol. Cytotoxicity was a primary concern because vitrification solutions have high cryoprotectant concentrations and the constructs are exposed to these compounds for extended periods during load/unload steps. A strategy that used lower cryoprotectant concentrations for load/unload steps while the vitrification solution would be at higher concentration (i.e., load/unload with VS55 but vitrify constructs in VS70) was pursued. In this way, the constructs were exposed for extended periods to a lower overall cryoprotectant concentration and only briefly exposed to the full-strength solution during vitrification. Constructs were vitrified in either VS55 or VS70 using load/unload solutions that were either based on the full-strength vitrification solution or were based on a solution that was lower in overall cryoprotectant concentration. Viability was measured upon rewarming and this strategy proved to be very effective and provided good viability right after rewarming but also sustained viability for 2 days post-rewarming (Figure 5). Viability among the various combinations was very similar right after rewarming, but viability at 1 and 2 days post-rewarming was significantly better for the combination where solutions with lower cryoprotectant loading concentrations than the vitrification solution were used (*p* < 0.0001, Figure 5).

Evaluation of other vitrification solutions (Table 1) was carried out, and at the same time a shift was made from using glass vials that held one construct to the use of a deep-well plate that could potentially hold 24 individual constructs at a time. This change in container from glass to plastic would allow for the simultaneous vitrification of several constructs at once, but there was the possibility that changing from a glass vial to a plastic deep-well plate could change the dynamics of cooling and warming which could have an impact on construct viability. This was not the case, and in fact, the constructs vitrified in the deep-well plate were viable, both after rewarming and over several days post-rewarming—viability was better than when constructs were vitrified in vials (Table 2). Several different vitrification solutions have been investigated using different concentrations of load/unload solutions. The four best combinations that were observed are highlighted blue in Table 2.

**Figure 6:**
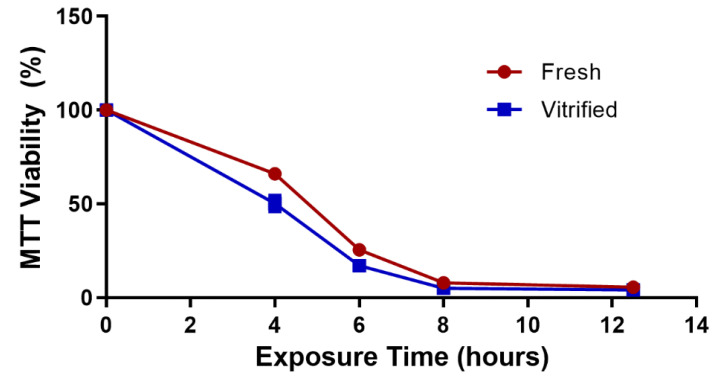
Viability of fresh and vitrified EpiDerm constructs after exposure to Triton-X100. Fresh constructs were assessed immediately, vitrified constructs were preserved using DP6 for the load/unload steps (5 min) and VS70 for vitrification. Viability was measured using the MTT assay and is the mean (±SEM) of ≥12 replicates. No significant differences were observed by one-way ANOVA with Tukey’s multiple comparison test.

Comparison of fresh and vitrified EpiDerm constructs after exposure to 1% Triton-X100 for up to 12.5 h followed by measurement of viability using the MTT assay was performed for constructs that were vitrified using the DP6/VS70 solution combination. The results with our optimized vitrification protocol were similar to the fresh control (Figure 6). Significant differences were not observed between fresh and vitrified constructs at any of the timepoints tested demonstrating that vitrified constructs react in a similar manner as untreated constructs when challenged with a potentially toxic substance.

**Figure 7:**
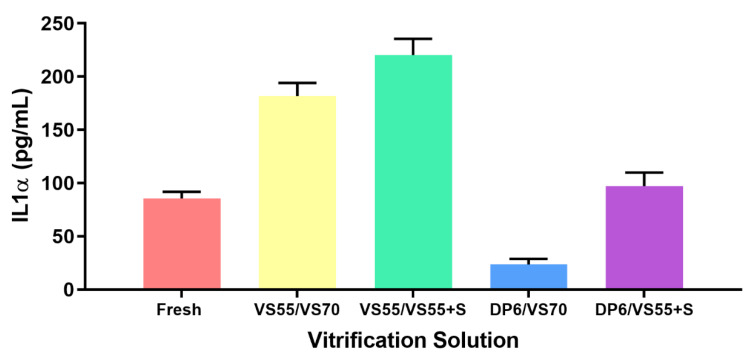
Comparison of IL-1α release for fresh and vitrified EpiDerm constructs. Constructs were loaded and vitrified with the indicated solution combinations. Upon rewarming the supernatants from each construct were retained and IL-1α was measured by ELISA. Values are the mean (±SEM) of ≥3 replicates. Statistical analysis performed by one-way ANOVA and Tukey’s multiple comparison test one-way ANOVA with Tukeys multiple comparison test.

Additional testing was carried out evaluating the release of IL-1α as compared to fresh controls. The cytokine IL-1α is constitutively expressed in many cell types and is considered a trigger for the induction of inflammation due to stress or injury. Cells that are injured or have a compromised plasma membrane may leak IL-1α and its presence outside the cell can facilitate an inflammatory response [47,48]. IL-1α was measured here as an indicator of the general health of the cells in constructs that had been vitrified as compared with fresh controls. Several vitrification solutions were tested (Figure 7). It is expected that cells within the constructs undergoing vitrification will endure some stress and potential injury from the preservation process. The goal is to minimize and/or eliminate cell injury/stress so that a construct can be used for other purposes. Some IL-1α release was observed with the fresh constructs that had not been treated. No significant differences were observed between fresh and constructs vitrified in either VS55/VS70 or DP6/VS55+sucrose. Significant differences were observed (*p* < 0.01) with DP6/VS70 and VS55/VS55+sucrose. Constructs vitrified in DP6/VS70 demonstrated a lower IL-1α release than the fresh control while the combination of load/unload with VS55 and vitrification with VS55 plus sucrose demonstrated higher IL-1α release. The amount of IL-1α measured from constructs that had been vitrified was dependent on the load/unload vitrification solution combination used. In other words, constructs where a lower concentration of cryoprotectants was used to load/unload the constructs demonstrated less IL-1α release than constructs that used load/unload solutions that were more equivalent in cryoprotectant concentration to the actual vitrification solution, suggesting that reducing exposure to higher concentrations of cryoprotectants reduces potential injury to the cells; this is likely due to cytotoxicity.

**Figure 8:**
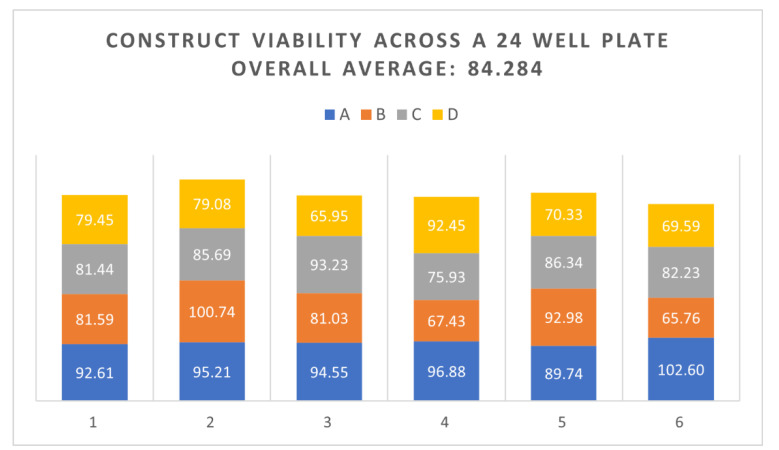
A full plate of EpiDerm constructs, 24, were vitrified and rewarmed using DP6/VS70. Viability was measured immediately after rewarming using the resazurin assay.

Having shifted to using deep-well plates for vitrifying constructs, experiments were performed that vitrified from 4–24 constructs at a time with maintenance of construct viability (see Table 2). Constructs are being used more and more for toxicity testing and the demand for tissue constructs for research continues to rise. The ability to vitrify multiple constructs at once would allow the development of a high throughput or automated process for preserving constructs that could streamline the use of such constructs in research as outlined in the introduction. Experiments vitrifying 24 constructs using a deep-well plate were carried out. Constructs were loaded/unloaded using DP6 and then vitrified with VS70 (Figure 8). Construct viability was measured after rewarming. As observed in Figure 8, construct viability across the plate was high and ranged from 65 to 102% of untreated controls. Overall, the average viability for all of the constructs in plates was 84%.

**Figure 9:**
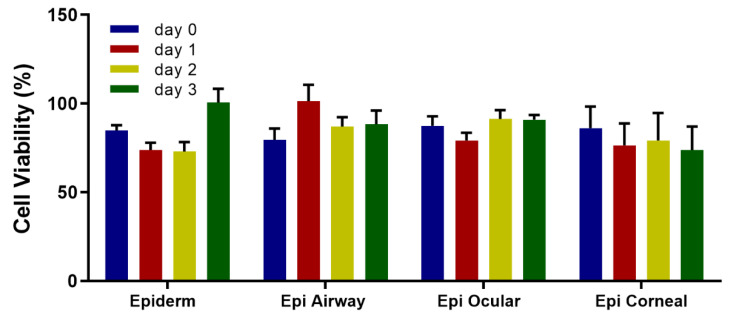
Viability of several constructs after vitrification in vials and a deep-well plate. The indicated constructs were vitrified with VS55/VS70. After rewarming, viability was measured using the resazurin assay and is the mean(±SEM) of ≥8 replicates.

**Figure 10:**
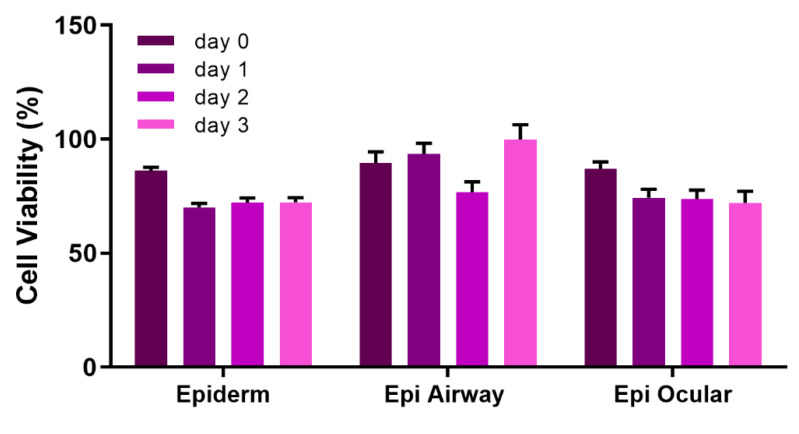
Viability after storage of various constructs. The indicated constructs were vitrified with DP6/VS70 in a deep-well plate and stored at <−135 °C in a vapor phase nitrogen storage freezer from 2–7 months. EpiDerm—6 months; Epi Airway—7 months; Epi Ocular—2 months. Viability was measured using the resazurin assay and is the mean (±SEM) of 8–12 replicates.

All of the above experiments were performed using the EpiDerm construct from MatTek. In order to test the robustness of our protocol, three other epithelial construct types (Epi Airway, Epi Ocular and Epi Corneal) were vitrified using a deep-well plate. These constructs responded well to being vitrified, and their viability was maintained upon rewarming for several days post-rewarming (Figure 9). While the constructs could be vitrified successfully in glass vials (data not shown), our observation was that their viability was more consistent when vitrified in the deep-well plate. Furthermore, constructs could be vitrified and stored for up to 7 months in a vapor phase nitrogen storage freezer (CryoPlus4, Thermo Fisher, Waltham, MA, USA) using the deep-well plate (Figure 10). Each construct was vitrified using DP6/VS70 and stored in a mechanical freezer at >−135 °C. Upon rewarming, the constructs demonstrated good viability immediately after rewarming (~>85%) that was sustained for several days post-warming (~>70%).

## 4. Discussion

Research into protocols for the preservation of cells and tissue constructs for regenerative medicine applications are limited, but the demand for such protocols is high as more and more cell- and tissue-based therapies are developed for drug and chemical testing or clinical use. Some researchers have focused on preservation strategies, such as encapsulation, that can protect cells during the preservation process by limiting ice formation and exposure to CPAs [49,50,51,52,53]. Other studies are pursing the preservation of tissue spheroids and cellular sheets by vitrification for clinical use, in assay systems, or as precursors for clinical use such as hepatocyte spheroids that are being used to seed liver-assist devices [54,55,56,57,58,59]. Still others are developing preservation strategies for more complex samples, such as bioengineered constructs, native tissues and even organs [9,15,55,59,60,61,62]. At present, bioengineered products are generally made to order, so a lead time is required before they can be used. For clinical use, delays such as this could make the difference between a successful treatment outcome and failure. Vitrification preserves the sample without the formation of ice, which is particularly important for native tissue and three-dimensional constructs that have not only cells but an extracellular matrix forming a complex scaffold microenvironment, whose integrity must be protected if viability and function of the tissue or construct after preservation is to be maintained.

Considerable research has been carried out developing vitrification protocols for reproductive materials—embryos, oocytes and sperm. This is its own niche, where the samples are very small, single cells, cell suspensions or small cell clusters, and vitrification is performed in small volumes using cryovials or more commonly straws as containers [63,64]. Our research has been focused on vitrification of more complex samples, such as bioengineered constructs and native tissues. Vitrification is believed to work well when the volumes being used are small, the concentration of cryoprotectant is relatively high and cooling and warming rates are sufficiently fast to avoid the formation of ice. However, paradigms are shifting and recent research is demonstrating that these vitrification parameters can be expanded to accommodate larger and more complex samples. Some of these changes include adjustments to solution composition, specifically the addition of non-permeating CPAs, such as disaccharide sugars [65,66], vitrification using larger volumes (≥2 mL) and alternative warming strategies to prevent ice formation during rewarming, such as nanowarming - radiofrequency (RF) excitation of magnetic nanoparticles (mNPs) in the cryoprotectant solution [67,68].

**Figure 11:**
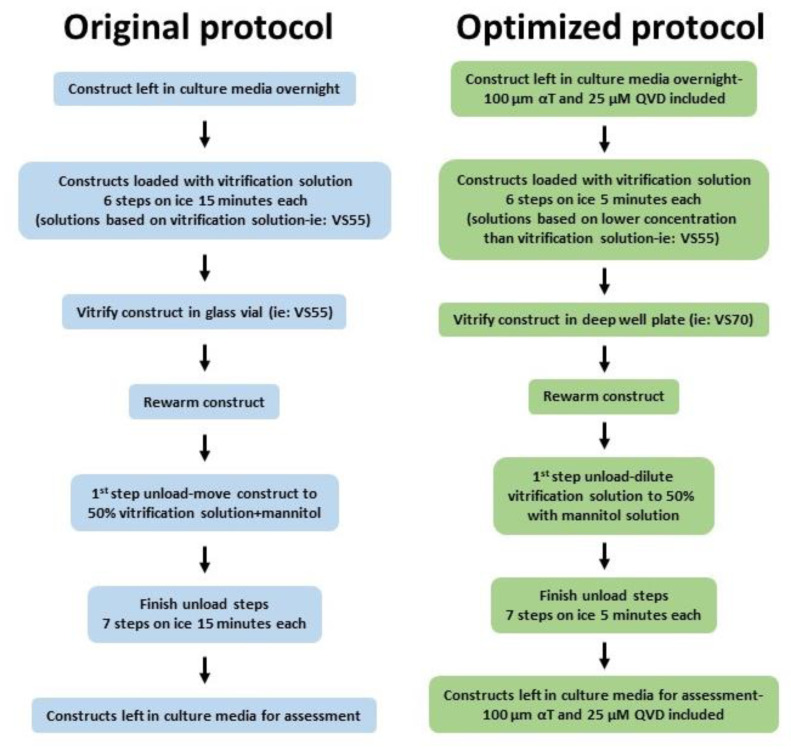
Vitrification protocols. Flow charts showing the steps for our optimized vitrification protocol for bioengineered constructs and the original vitrification protocol used with blood vessels.

In this study, initial experiments began by using a vitrification protocol that had already been proven successful in preserving blood vessels [18,19,30,31,32,33] based on the original parameters for vitrification (Figure 11, below). This protocol was then modified and optimized to maximize construct viability immediately after rewarming and for several days post-rewarming (Figure 11). It is not unusual for samples that have been cryopreserved to demonstrate good viability right after rewarming/thawing and then show a decrease in viability within the first 24 h in culture. This is likely due to the induction of apoptosis [10,49]. Therefore, maintenance of viability after rewarming with little if any decrease over several days is valuable. The quality of the tissue construct is maintained and allows for flexibility in utilization of the rewarmed constructs for experiments.

The optimized protocol included the following changes (Figure 11): (1) Several of the vitrification formulations that were assessed included the addition of non-permeating sugars—sucrose and trehalose. (2) Load/unload solutions that were at a lower concentration, then the vitrification solutions were used to reduce cytotoxicity from exposure to the CPAs. (3) Supplements were included prior to and after vitrification to facilitate viability of the construct. (4) Multiple constructs were vitrified in a multi-well plate as opposed to the one-construct-per-container scenario. Early experiments used individual constructs vitrified in glass vials. However, from a manufacturing perspective, the ability to vitrify multiple constructs at once would be more economical, and more amenable to future automation of the process. Deep-well plates made of polypropylene plastic that could hold 24 inserts were used. Each well can hold up to 10 mL of solution and each well has a round bottom that is exposed, not covered by a flat-bottom piece of plastic. These unique characteristics allowed for the placement of the insert into the bottom of each well, such that vitrification solution would cover the bottom of the insert using a relatively small volume, 600 uL. Then, a small volume, 200 uL, of vitrification solution was placed inside the insert to cover the construct prior to vitrification. Small volumes of CPA solution were used, and vitrification was successful even though it was accomplished in a plastic multi-well plate. Plastic is not considered a good conductor of heat as opposed to glass, and so it would be expected that cooling and warming rates would not be as fast as if a glass container were used potentially preventing vitrification of the CPA solution. In addition, these constructs were grown on a well insert that was also present within the well of the deep-well plate, adding more plastic architecture to the system that had to be cooled and warmed adequately for vitrification to be maintained. However, vitrification using the deep-well plate worked very well. Construct viabilities were as good as or better than vitrification using a glass vial (Table 2). Moreover, vitrification of 24 constructs at a time was achieved with maintenance of viability after rewarming and for several days post-rewarming, similar to viability measured using the glass vial (Figure 8). Optimization will likely be required to minimize variability between the constructs; however, the lowest viability measured is still considered adequate by the manufacturer of EpiDerm for toxicity testing purposes. Automation of the process will likely keep viability high and sustained, while decreasing variability across the plate.

The versatility of this protocol means that it is potentially applicable to other types of tissue, both natural and bioengineered. Three other constructs besides EpiDerm were evaluated: Epi Airway—derived from human tracheal/bronchial epithelium; Epi Ocular—derived from human epidermal keratinocytes; Epi Corneal—derived from human corneal epithelial cells. All were vitrified and demonstrated good viability for several days post-rewarming after short- and long-term storage at <−135 °C (Figure 9 and Figure 10).

## 5. Conclusions

In conclusion, a new, unique, robust protocol for the preservation of tissue-engineered constructs has been developed, which has proven successful with several different bioengineered epithelial tissue construct types. Not only is viability maintained for several days after rewarming, but it was shown that multiple constructs could be preserved at one time, suggesting that automation of this process may be successful and amenable to many tissue types, whether established on a well insert or not. The use of higher concentrations of cryoprotectants may also permit the cost-effective transport of tissue equivalents on dry ice, because some of these formulations do not demonstrate ice nucleation at temperatures warmer than the formulation glass transition [65,68]. Future experiments are planned to explore automation of this vitrification protocol as well as preserving bioengineered constructs derived from other tissue types.

## 6. Patents

Preservation of Natural and Bioengineered tissues and Methods of Storing and Transport—submitted November 2021.

## Figures and Tables

**Table 1:** Vitrification solution formulations.

Vitrification Solution	Dimethyl Sulfoxide (DMSO)	1,2-Propanediol (PD)	Formamide (FD)	Concentration (M)
VS49	2.75	2.0	2.75	7.5
VS55	3.1	2.2	3.1	8.4
VS70	3.88	2.75	3.88	10.7
VS83	4.65	3.3	4.65	12.6
DP6	3.0	3.0	---	6.0
DP7	3.5	3.5	---	7.0
DP8	4.0	4.0	---	8.0

**Table 2:** Construct viability using different loading and vitrification solutions *.

Load/Unload Solution	Vitrification Solution	Container	Viability Day 0	Viability Day 2
VS55	VS55	vial	90.2 ± 1.4	22.6 ± 1.8
VS55	VS55 + 15% glycerol	vial	84.7 ± 10.6	26.7 ± 3.6
VS55	VS49 + 0.6 M sucrose	vial	73.9 ± 3.0	44.1 ± 2.9
VS55	VS70 + 0.6 M sucrose	vial	59.1 ± 3.6	24.7 ± 3.2
VS55	VS70 + 0.6 M trehalose	vial	54.0 ± 3.8	15.9 ± 5.0
VS70	VS70	vial	98.7 ± 9.2	13.6 ± 5.2
DP6	DP6 + 0.6 M sucrose	vial	70.8 ± 6.2	16.9 ± 5.5
VS83	VS83	vial	79.4 ± 4.2	0.6 ± 0.3
VS55	VS55 + 0.6 M sucrose	vial and plate	93.5 ± 8.6	73.5 ± 3.6
VS55	VS70	vial and plate	86.4 ± 6.7	80.9 ± 7.7
VS49	VS70	plate	80.3 ± 2.8	25.5 ± 2.9
VS49	VS55 + 0.6 M sucrose	plate	81.2 ± 4.4	25.5 ± 3.6
DP6	VS55 + 0.6 M sucrose	plate	75.4 ± 10.3	52.3 ± 14.1
DP6	VS55 + 0.6 M sucrose + trehalose	plate	94.3 ± 3.1	62.8 ± 2.5
DP6	VS70	plate	86.2 ± 8.4	73.9 ± 8.6
DP6	DP7 + 0.6 M sucrose	plate	76.2 ± 7.1	21.8 ± 3.7
DP6	DP7 + 0.6 M trehalose	plate	84.1 ± 5.6	36.3 ± 8.3
DP6	DP7 + 0.6 M sucrose + trehalose	plate	89.0 ± 6.0	67.9 ± 9.2
DP6	DP8 + 0.6 M sucrose	plate	89.2 ± 4.4	23.0 ± 3.1
DP7	DP7 + 0.6 M sucrose	plate	97.6 ± 3.6	75.2 ± 4.9

* Solution composition listed in Table 1.

## Data Availability

Not applicable.
